# 

**Published:** 1974-10

**Authors:** 


					VJ|
i)
I?9
ass
BRISTOL
MEDICO
CHIRURGICAL
JOURNAL
^NAriONAL^
LIBRARY, \
DEC Or 1375 ^
w F
MEDICINE
OCTOBER 1974
V
VOL. 89 (iv) No. 332

				

